# Relationship between neutrophil to lymphocyte ratio and diabetic peripheral neuropathy: a systematic review and meta-analysis

**DOI:** 10.1186/s40001-023-01479-8

**Published:** 2023-11-16

**Authors:** Armin Rezaei Shahrabi, Gabrielle Arsenault, Seyed Ali Nabipoorashrafi, Brandon Lucke-Wold, Shirin Yaghoobpoor, Fatemeh Zari Meidani, Rahem Rahmati, Arshin Ghaedi, Shokoufeh Khanzadeh

**Affiliations:** 1 Department of Pharmacy, Damghan Branch, Islamic Azad University, Damghan, Iran; 2 Department of Neurosurgery, University of Florida, Gainesville, USA; 3 Endocrinology and Metabolism Research Center (EMRC), School of Medicine, Vali-Asr Hospital, Tehran, Iran; 4 Student Research Committee, Faculty of Medicine, Shahid Beheshti University of Medical Sciences, Tehran, Iran; 5 Students Research Committee, Shahrekord University of Medical Sciences, Shahrekord, Iran; 6 Student Research Committee, School of Medicine, Shiraz University of Medical Sciences, Shiraz, Iran; 7 Trauma Research Center, Shahid Rajaee (Emtiaz) Trauma Hospital, Shiraz University of Medical Sciences, Shiraz, Iran; 8 Tabriz University of Medical Sciences, Tabriz, Iran

## Abstract

**Background:**

The present study aims to review the existing scientific literature on the role of neutrophil to lymphocyte ratio (NLR) in diabetic peripheral neuropathy (DPN) to perform a meta-analysis on the available data.

**Methods:**

The electronic repositories Web of Science, PubMed, and Scopus were systematically explored starting from their establishment up until June 9, 2022.

**Results:**

Fifteen articles were included in the meta‐analysis after multiple screening according to the PRISMA guidelines. The combined findings indicated that individuals with DPN had higher levels of NLR in comparison to those without DPN (SMD = 0.61; CI 95% = 0.40–0.81, *p* < 0.001). In the subgroup assessment based on ethnicity, it was observed that diabetic patients with DPN exhibited increased NLR levels in contrast to those without DPN in studies conducted in India (SMD = 1.30; CI 95% = 0.37–2.24, *p* = 0.006) and East Asia (SMD = 0.53; CI 95% = 0.34–0.73, *p* < 0.001) but not in studies conducted in Turkey (SMD = 0.30; CI 95% = − 0.06–0.67, *p* = 0.104) and Egypt (SMD = 0.34; CI 95% = -0.14–0.82, *p* = 0.165). The pooled sensitivity of NLR was 0.67 (95% CI = 0.49–0.81), and the pooled specificity was 0.70 (95% CI, 0.56–0.81). The pooled positive likelihood ratio, negative likelihood ratio, diagnostic odds ratio (DOR) of NLR were 2.30 (95% CI = 1.71–3.09), 0.45 (95%CI = 0.30–0.67), and 5.06 (95% CI = 3.16–8.12), respectively.

**Conclusion:**

NLR serves as a distinct marker of inflammation, and its rise in cases of DPN suggests an immune system imbalance playing a role in the development of the disease.

**Supplementary Information:**

The online version contains supplementary material available at 10.1186/s40001-023-01479-8.

## Background

Type 2 diabetes mellitus, which is an age-related disorder, is characterized by hyperglycemia that results in chronic low-grade inflammation. This chronic inflammation leads to various complications and, most often, diabetic peripheral neuropathy (DPN) [1, 2]. DPN is a significant global public health issue [3]. It is one of the key factors contributing to morbidity and rising mortality [4]. Research conducted by the American Diabetes Association (ADA) revealed that around 26.4% of individuals with type 2 diabetes experience the challenge of painful DPN. Moreover, a substantial portion, potentially up to 50%, of those affected by DPN might not exhibit any noticeable symptoms [5]. The diagnosis of DPN relies on both a nerve conduction study and clinical examination [5]. DPN may progress to diabetic foot lesions like infection, gangrene of the feet, ulcers, and amputation [6]. These late consequences are related with increased mortality and worse quality of life, as well as a large cost to healthcare systems [7]. As a result, finding a reliable biomarker for the early detection of DPN is critical.

The association between diabetic neuropathy and inflammation is well established [8]. Besides, NLR is increased in conditions that characterized with inflammation, such as gastrointestinal diseases [9], cardiac conditions [10], thyroiditis [11], and other thyroid conditions [12], irritable bowel disease [13], trauma [14], and Covid-19 infection [15]. Thus, analyzing the relationship between NLR and diabetic neuropathy makes sense. Recent studies have reported significant elevated Neutrophil to lymphocyte ratio (NLR) levels in DPN patients compared to T2D patients without DPN [3, 5, 16–28]. NLR has been used as a novel biomarker and diagnostic tool to test for chronic inflammation. NLR has been used to indicate the rise of comorbidities accompanying cardiac diseases and may help reveal clinical outcomes following stroke, such as post-stroke infection mortality rates [29–31]. This meta-analysis aimed to analyze and extract the data from previously published literature to identify the changes of NLR in patients with DPN.

## Methods

### Search strategy

**Fig. 1:**
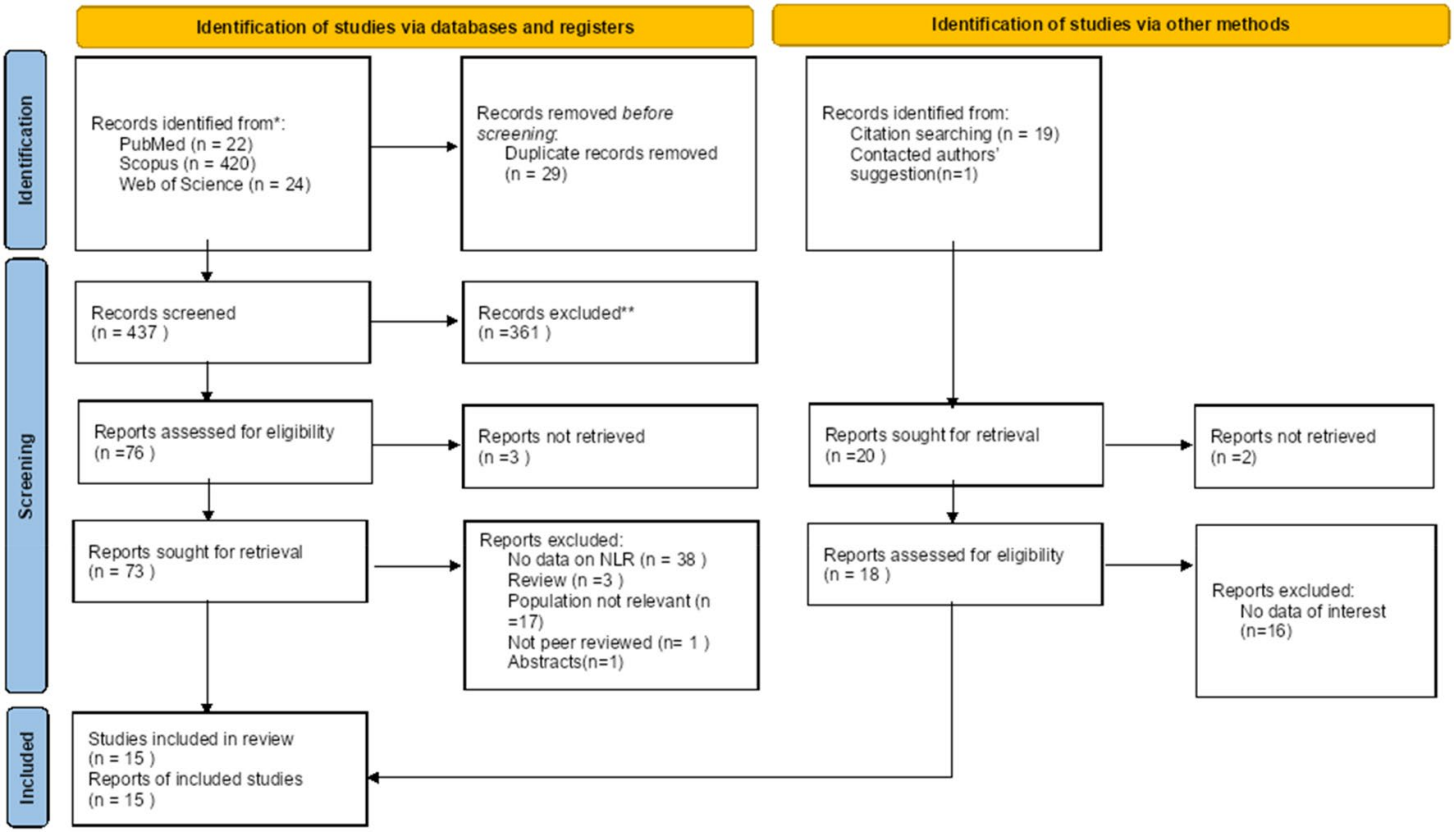
PRISMA 2020 Flow diagram for new systematic reviews which includes searches of databases, registers and other sources

We performed a comprehensive systematic review and meta-analysis, adhering to the Preferred Reporting Items for Systematic Review and Meta-analyses (PRISMA) guidelines, to gather all published materials, including preprints and non-traditional sources like grey literature [32] **(**Fig. 1**).**

Two reviewers, unaware of journal and author information, carried out an unbiased systematic literature search in databases of PubMed, Web of Science, and Scopus using the following strategy: ("Neutrophil to lymphocyte ratio" OR NLR) AND "diabet*" AND "neuropathy".

The final search update occurred on June 9, 2022. Our search approach had no limitations on language or publication year. Additionally, we examined the reference lists of pertinent reviews and articles to locate potential eligible studies. We also checked the Prospero Register for information about unpublished and ongoing studies. To uncover grey literature and additional pertinent studies, we conducted a supplementary, informal search on Google Scholar as a secondary database.

### Inclusion and exclusion criteria

We employed the PICOS (population, intervention, control, outcomes, and study design) principle to determine eligible studies, ensuring a systematic exploration of existing literature. The following inclusion criteria were outlined:

- Population: Patients with type 2 diabetes mellitus who developed DPN
- Intervention. NLR
- Control. Patients with type 2 diabetes mellitus without DPN
- Outcomes. The diagnostic performance of NLR in DPN
- Study Design. We anticipated that the papers would adhere to a case–control or cross-sectional research design. However, our search was not restricted to any specific research methodology.

We excluded reviews, comments, case reports, case series, editorials, letters, papers with insufficient data, duplicate items, and irrelevant papers.

### Data extraction and quality assessment

Two authors independently scrutinized the titles and abstracts of the acquired articles. Subsequently, the same two authors individually assessed the full texts of pertinent articles for eligibility. In case of disparities between reviewers during both stages, a third independent author intervened to reach a resolution. The writers extracted the following items from each study: publication year, study design, the first author’s name, country of the study, the number of cases and controls, mean ± SD or other data (median, interquartile range, and/or range) (IQR) of NLR of participants in each study. Any inconsistencies were resolved through dialogue involving a third author.

Two authors conducted an evaluation of the included studies' quality, separately. They used the Newcastle–Ottawa Scale [33] for this assessment, which consists of three parts: selection (4 items), comparability (2 items), and exposure (3 items), resulting in a potential score range of 0 to 9. If there were any differences of opinion, a third author acted as a mediator to reach a consensus.

### Statistical analysis

The NLR level was presented as a Standardized Mean Difference (SMD) along with a 95% confidence interval (CI). To compute the mean and standard deviation from the median, sample size, and either the range or interquartile range (IQR), the techniques outlined by Wan et al. were employed [34]. Heterogeneity among the outcomes of the studies was evaluated through both the chi-squared (*χ*^2^) test and the *I*^2^ statistic. The *χ*^2^ test was employed to determine if there was heterogeneity present, while the *I*^2^ statistic quantified the degree of inconsistency across the studies. When the *I*^2^ value exceeded 75% and the *p*-value from the *χ*^2^ test was less than 0.05, it indicated significant heterogeneity in the results. In such instances, a random-effects model was used for the meta-analysis of the diverse outcomes. Otherwise, a fixed-effect model was utilized.

To assess the diagnostic value of NLR for DPN, the "metandi" command was employed to analyze the sensitivity, specificity, Summary Receiver Operating Characteristic (SROC) curve, negative likelihood ratio, Diagnostic Odds Ratio (DOR), and positive likelihood ratio.

To identify any potential publication bias, both Egger's linear-regression test and a Funnel plot were utilized. A *P*-value of less than 0.05 in these tests indicated significant publication bias. All statistical analyses were carried out using STATA 12.0 software from Stata Corporation in College Station, TX, USA. A *P*-value ≤ 0.05 was considered indicative of statistical significance.

## Results

### Search and selection of literature

Initially, 486 records were identified through a combination of database searches and a manual review of article citations. Following the removal of duplicate entries and irrelevant records, a total of 15 studies [3, 5, 16–28] were deemed suitable for inclusion in the systematic review and subsequent meta-analysis. These studies collectively encompassed 4575 patients diagnosed with type 2 diabetes, of which 1708 individuals developed DPN.

The step-by-step procedure of including and excluding studies is elaborated in the PRISMA flowchart, available in Fig. 1. Additionally, the PRISMA checklist pertinent to this research is furnished in Additional file 1.

### Characteristics of the included studies

This meta-analysis included 15 studies, of whom seven were conducted in East Asia [3, 17, 18, 20, 26–28], three in Egypt [5, 16, 22], three in India [21, 23, 25], and two in turkey [19, 24]. Regarding the language used in the documents, they were all authored in the English language. All of them were retrospective studies. The general features of the studies, as well as their quality ratings, are shown in Table 1. In total, 14 research compared NLR levels of diabetic patients with and without DPN [5, 16–28] and five studies reported diagnostic value of NLR in DPN, based on ROC curve analysis[3, 16, 18, 23, 26]. NOS score of included studies ranged between 6 and 7.

**Table 1:** General characteristics of included studies

First author	Year	Country	Ethnicity	Design	NLR cut-off	SEN	SP	Non-DPN group		DPN group		NOS score
								N	NLR	N	NLR	
Moursy	2015	Egypt	Egyptian	Prospective	–	–	–	27	1.74 ± 0.46	81	2.61 ± 1.60	6
Liu	2017	China	East Asian	Retrospective	1.7	63	72	233	–	278	–	8
Xu	2017	China	East Asian	Prospective	2.13	81	48	397	2.18 ± 0.61	160	2.58 ± 0.50	8
Demirdal	2018	Turkey	Turkish	Retrospective	–	–	–	261	6.60 ± 5.80	19	9.80 ± 11.50	7
Mineoka	2018	Japan	East Asian	Prospective	–	–	–	203	1.92 ± 0.76	132	2.13 ± 0.77	8
Ranjith	2018	India	Indian	Prospective	2.26	88	57	63	1.96 ± 0.60	55	2.60 ± 0.76	6
Senyigit	2018	Turkey	Turkish	Prospective	–	–	–	95	2.32 ± 1.29	30	2.49 ± 1.29	7
Yan	2019	China	East Asian	Prospective	–	–	–	1129	3.10 ± 2.58	213	4.40 ± 4.00	8
MK	2020	India	Indian	Prospective	–	–	–	86	1.91 ± 0.76	18	2.57 ± 1.90	7
Raya	2020	Egypt	Egyptian	Prospective	1.84	57	88	30	1.92 ± 0.89	30	2.44 ± 1.11	6
Wadhwani	2020	India	Indian	Prospective	–	–	–	78	1.49 ± 0.54	22	3.14 ± 1.09	7
Zhao	2022	China	East Asian	Prospective	–	–	–	79	1.76 ± 0.63	481	2.20 ± 0.94	7
AbdelAziz	2021	Egypt	Egyptian	Prospective	–	–	–	15	1.57 ± 0.84	45	1.43 ± 0.62	6
Chen,M	2021	China	East Asian	Prospective	2.48	38	79	81	1.80 ± 0.70	74	2.00 ± 0.84	7
Chen,Y	2021	China	East Asian	Prospective	–	–	–	90	1.93 ± 0.66	70	2.66 ± 0.68	7

*N* Number, *NLR* Neutrophil to lymphocyte ratio, *R* Retrospective, *P* Prospective, *NOS* Newcastle–Ottawa Scale, *DPN* Diabetic peripheral neuropathy

### Difference in NLR level between diabetic patients with and without DPN

**Fig. 2:**
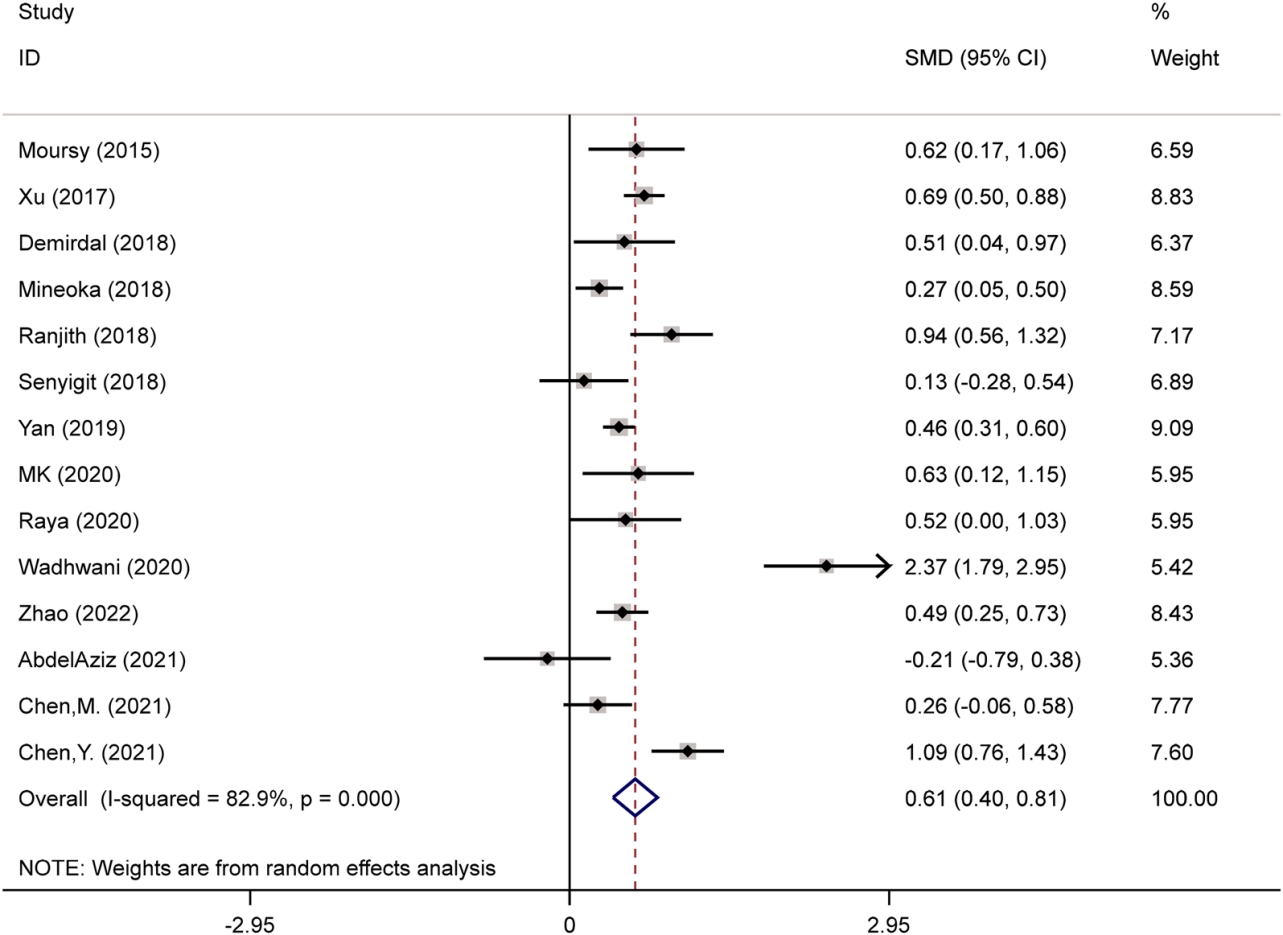
Meta-analysis of differences in NLR level between diabetic patients with and without DPN

Because the included articles were statistically heterogeneous [5, 16–28] (*I*^2^ = 82.9%, *p* < 0.001) the random-effect model was utilized in the analysis (Fig. 2). The pooled results indicated that diabetic individuals with DPN had higher levels of NLR when compared to those without DPN (SMD = 0.61; CI 95% = 0.40–0.81, *p* < 0.001).

**Fig. 3:**
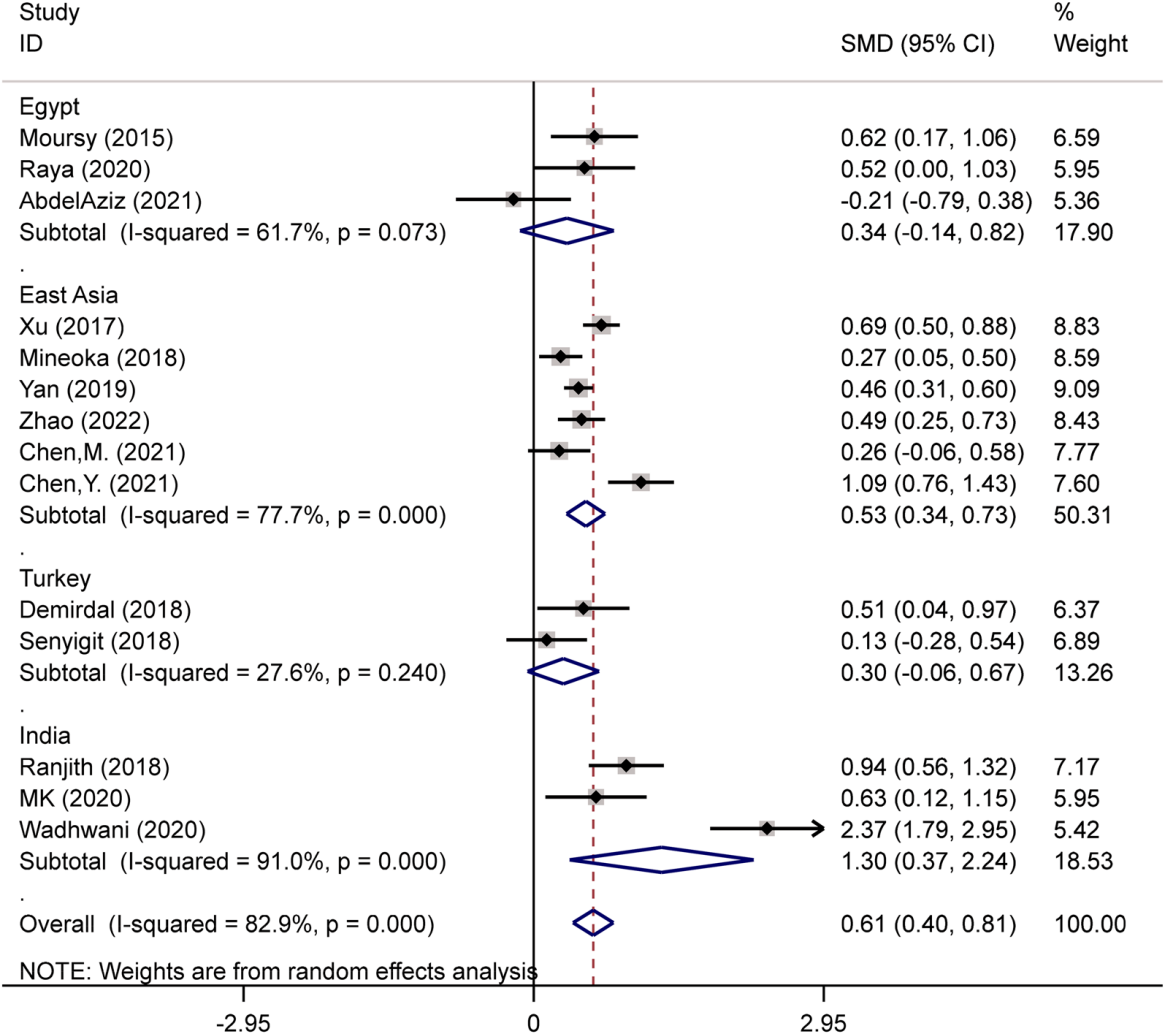
Subgroup analysis of differences in NLR level between patients with and without DPN, according to study location

In the subgroup examination based on ethnic background, it was observed that diabetic patients having DPN demonstrated higher NLR levels in contrast to those lacking DPN in studies carried out in India (SMD = 1.30; CI 95% = 0.37–2.24, *p* = 0.006) and East Asia (SMD = 0.53; CI 95% = 0.34–0.73, *p* < 0.001) but not in studies conducted in Turkey (SMD = 0.30; CI 95% = − 0.06–0.67, *p* = 0.104) and Egypt (SMD = 0.34; CI 95% = -0.14–0.82, *p* = 0.165) (Fig. 3).

### Diagnostic value of NLR in DPN

**Fig. 4:**
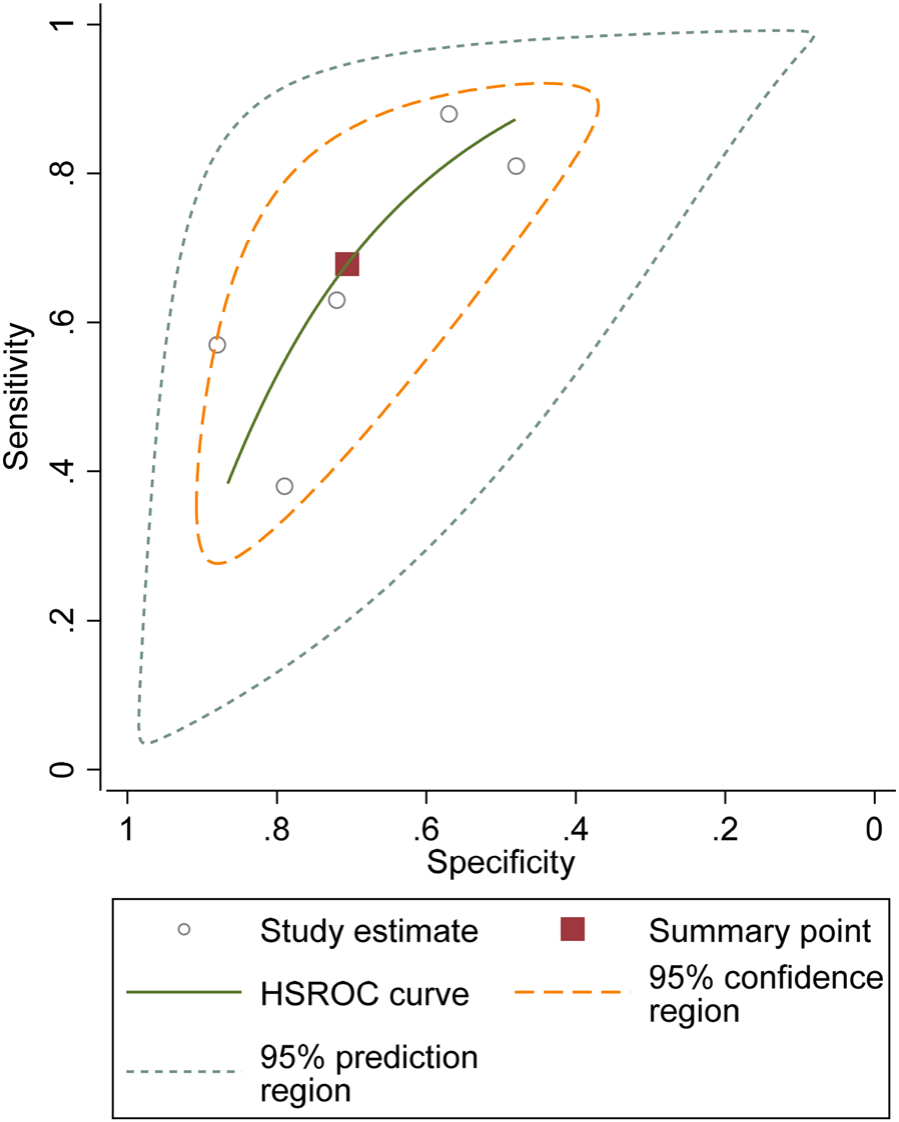
SROC curve of included studies assessing diagnostic value of NLR for DPN

SROC curve of five studies [3, 16, 18, 23, 26] assessing diagnostic value of NLR for DPN showed that the pooled sensitivity of NLR was 0.67 (95% CI = 0.49–0.81), and the pooled specificity was 0.70 (95% CI, 0.56–0.81). The pooled positive likelihood ratio, negative likelihood ratio, diagnostic odds ratio (DOR) of NLR were 2.30 (95% CI = 1.71–3.09), 0.45 (95% CI = 0.30–0.67), and 5.06 (95% CI = 3.16–8.12), respectively (Fig. 4).

### Publication bias

**Fig. 5:**
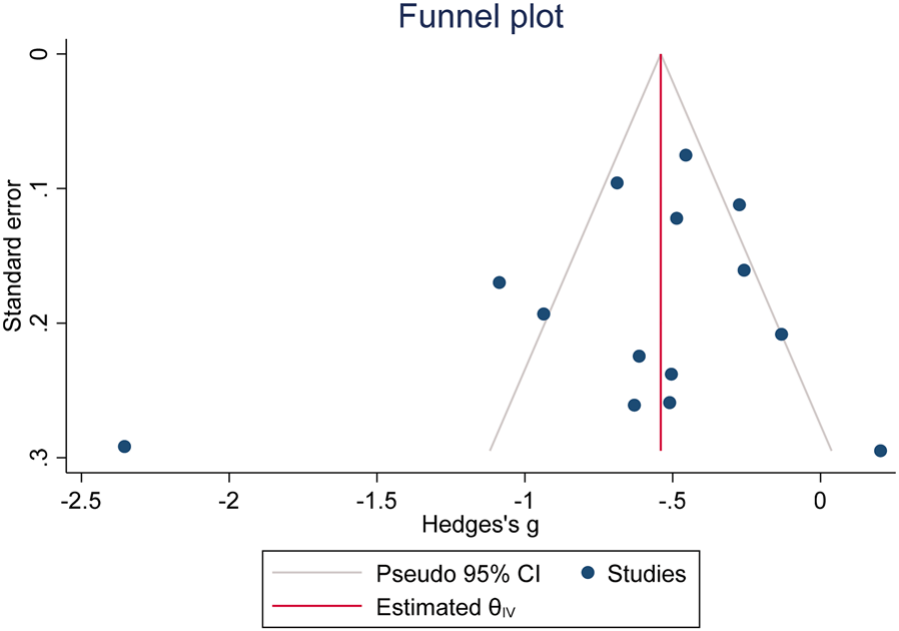
Funnel plot assessing publication bias

As depicted in Fig. 5, there was no indication of publication bias within the studies that were included (Egger’s test *p* = 0.42).

## Discussion

The current study found significantly increased NLR levels in T2D patients with DPN compared to T2D patients without DPN. The meta-analysis involving 14 studies displayed statistically significant differences between the two groups of patients [5, 16–28]. A second meta-analysis was conducted following the initial meta-analysis, dividing the studies into subgroups according to a geographical region to account for the high heterogeneity. This subgroup analysis consisted of four groups: Egypt, East Asia, Turkey, and India. Of the four groups only East Asia and India reported statistical significance. This study did produce clear insights and results displaying a moderate effect. The subgroup meta-analysis shed light on India being the possible source of most heterogeneity and a source of a wide confidence interval; with this data, the studies from India may have a lower certainty than the four other subgroups.

One of the most prevalent consequences of diabetes is diabetic DPN. DPN has a gradual onset, a sluggish progression, with symptoms such as symmetrical numbness, pain, and paresthesia in the early stages, but ulcers and gangrene of the foot in the latter stages [35]. As a result, preventing DPN and detecting it early are critical to enhancing diabetic patients' quality of life. Previous studies have reported increased NLR levels being significantly associated with hypertension, hyperglycemia, DPN, cardiac disease comorbidities, and high NLR levels may be a reliable predictive biomarker of developing and early-stage diabetic nephropathy [36].

WBC count and its components are well-known inflammatory indicators that are easy to obtain and measure [37]. However, whether using WBC, neutrophil, or lymphocyte counts to make a diagnosis, there are a number of biases to consider. Nevertheless, because NLR is a dynamic parameter, it has a better predictive value and when compared to other leukocyte indicators (such as neutrophil, lymphocyte, and total leucocyte count), NLR is less affected by physiological, pathological, and physical variables [38–40]. NLR level testing is an inexpensive diagnostic tool that can help clinicians predict their patients’ outcomes and help create treatment plans. Further studies are needed to continue analyzing NLR levels significance in the prediction of diabetic complications prognosis.

An SROC curve was used as a diagnostic tool to evaluate the accuracy of using NLR levels to clinically test for DPN in T2D patients and resulted in statistically significant results. The SROC curve displayed that if a patient received a positive test, they were 2.32 times more likely to develop DPN than a patient without diabetes, and if the test was negative, the patient was 0.43 times less likely to create DPN. The AUC of the SROC curve was 79% and demonstrated a moderate to a high level of accuracy in detecting DPN from NLR levels. The SROC curve is a beneficial source of information, deriving from a summary of several conducted studies examining NLR levels and their association with DPN by pooling an average sensitivity, and specificity, and defining a summary ROC curve. This summary allows for a more accurate interpretation of a wide variety of data supporting the association of NLR and DPN.

Our results and the literature review suggest that NLR can be a valuable biomarker in clinical practice due to numerous reasons such as: (1) NLR may serve as a diagnostic tool for DPN and it has shown clinical use in evaluating general health and possible risk factors associated with numerous medical diseases, including DPN. (2) A high NLR indicates an unbalanced immune response, which could lead to the nerve damage seen in DPN. (3) Studies have demonstrated that having a higher NLR increases the likelihood of complications in a variety of chronic illnesses, like diabetes [41]. Individuals exhibiting a high NLR may be at an increased risk of acquiring more severe DPN or encountering complications like foot ulcers or diabetic retinopathy [42, 43]. (4) NLR may be employed as a simple and low-cost marker to track the course of DPN. A rising NLR over time may signal a deteriorating inflammatory response and the necessity for more aggressive treatment. (5) When deciding on the best treatment approach for DPN, physicians might consider the NLR as part of the overall evaluation. It may assist in guiding treatment decisions and intensifying efforts for individuals who are at increased risk owing to increased NLR values. Endothelial injury, microvascular dysfunction, metabolic problems, oxidative stress, aberrant cytokines, and immunological variables all contribute to the development of DPN, with inflammatory injury playing a key role. Microcirculation problems can be caused by chronic hyperglycemia. Vascular pathological alterations such as vascular endothelial cell proliferation, thickening of the microvascular basement membrane, and hyaline degeneration can all lead to direct lumen narrowing. The loss in blood supply to local tissues is exacerbated by increased blood viscosity and blood flow disturbances. Ischemia and hypoxia of nerve tissues are caused by this process, which stimulates the production of cytokines and worsens inflammatory damage [14].

Higher NLR is made up of two primary components of a chronic inflammatory disease (high neutrophil and low lymphocyte). A high neutrophil count indicates that a damaging nonspecific inflammatory process is developing. A low lymphocyte count indicates insufficient regulation of immunologic processes as well as a quiescent immune system. As a result, increased NLR can indicate the immune system's functioning condition during chronic inflammation [39].

In vivo, the NLR represents the equilibrium between neutrophils and lymphocytes. Inflammatory reactions are tightly linked to neutrophils, and immunological regulatory pathways are reflected in lymphocytes [14, 44]. Neutrophils can represent systemic inflammation, as well as innate immune responses (mediated by neutrophils) and lymphocytes can represent adaptive immunological responses (mediated by lymphocytes) [45, 46]. Hyperglycemia-induced nonspecific inflammatory response may cause alterations in peripheral blood cell counts, which could explain the elevated NLR results.

Following an injury or infection, neutrophils are one of the first types of peripheral immune cells to arrive at the site of inflammation [2]. They play a vital role in initiating an immunological response, since they can generate both proinflammatory mediators and present antigen to T-cells [3]. The infiltration of immune cells both peripherally and centrally is a key mechanism underpinning the formation and maintenance of neuropathic pain in experimental nerve lesion models [47]. In spinal cord injury models, neutrophil migration into the CNS is well documented [48]. In a study, after 8 weeks of Streptozotocin-induced diabetic rats, Newton et al. found increased numbers of neutrophils and levels of L-selectin, an adhesion molecule necessary for neutrophil transmigration, in the lumbar spinal cord. These findings imply that spinal L-selectin dysregulation and neutrophil infiltration into the spinal cord may play a role in the development of painful DPN [49].

Lymphocytes represent the protective element of inflammation and immune regulatory pathways [50], which can explain higher NLR in T2D patients with DPN than those without. Also, alterations in the oxidative DNA damage of lymphocytes in T2D patients with DPN had been reported [51]. Reactive oxygen species (ROS) produced in vivo are thought to play a role in nerve injury [52, 53]. Poor glycemic control in diabetic patients results in chronic hyperglycemia. The oxidation of high glucose levels inside the cells enhances the generation of ROS and enhances oxidative stress [52]. The oxidation and change of the structure of cellular nucleic acids, proteins, and membrane lipids is caused by a rise in the production of ROS such as superoxide, hydrogen peroxide, and the hydroxyl radical. Kasznicki et al. [51] discovered that T2D patients' lymphocytes with and without distal symmetric polyneuropathy (DSPN) were more vulnerable to hydrogen peroxide-induced DNA damage. This finding could be due to a lack of antioxidant protection in diabetic patients, as well as a decrease in the levels of endogenous and exogenous free radical scavengers [54–56]. Also their study provided evidence that oxidative stress may be linked to the development of DSPN, since they found significantly higher levels of oxidative DNA damage in lymphocytes of T2D patients with concurrent DSPN compared to T2D patients without DSPN and control participants [51].

This study has several strengths. First, we gathered all available data on the association of NLR with DPN. To the best of our knowledge, this is the first meta-analysis in this context. According to previous reports, systemic disorders can fluctuate the level of inflammatory biomarkers which could compromise our results; however, most of the included studies excluded patients with such diseases (renal dysfunction, malignancy, steroid therapy, hepatic insufficiency, inflammatory diseases, hematologic disorders, and acute or chronic infections) to eliminate their effects. Obviously, including this exclusion rule among included articles might significantly improve the validity of our findings.

However, some limitations should be considered when interpreting the findings of our investigation. Despite the fact that this Meta-analysis was conducted using a random effect model and also conducting subgroup analysis, heterogeneity among the included studies still occurred. Differences in some features of the included studies, such as ethnicity, age, body mass index, and disease duration, could be potential sources of heterogeneity. Also, there were limited number of studies eligible to be included in our meta-analysis.

## Conclusion

In conclusion, the results of this meta-analysis support the significant higher levels of NLR among T2D patients with DPN than those without. Also, evaluating the accuracy of using NLR levels to clinically test for DPN in T2D patients showed significant results. Therefore, NLR could be utilized in clinics as a potential predictor to aid physicians in the detection of DPN among T2D patients. Further research is required to conduct meta-analysis with higher number of studies to attain more precise results. Also more research is needed to evaluate the potential association of NLR with DPN severity among T2D patients.

### Supplementary Information

**Additional file 1.** PRISMA 2020 checklist.

## Data Availability

All data generated or analyzed during this study are included in this published article.

## Abbreviations

NLR: Neutrophil to lymphocyte ratio
DPN: Diabetic peripheral neuropathy
DOR: Diagnostic odds ratio
PRISMA: Preferred Reporting Items for Systematic Review and Meta-analyses
PICOS: Population, intervention, control, outcomes, and study design
IQR: Interquartile range
SMD: Standardized mean difference
CI: Confidence interval
SROC: Summary receiver operating characteristic
ROS: Reactive oxygen species
DSPN: Distal symmetric polyneuropathy
